# Gentle Massage Improves Disease- and Treatment-Related Symptoms in Patients with Acute Myelogenous Leukemia

**DOI:** 10.4172/2167-0870.1000161

**Published:** 2014-03-17

**Authors:** Ann Gill Taylor, Audrey E Snyder, Joel G Anderson, Cynthia J Brown, John J Densmore, Cheryl Bourguignon

**Affiliations:** 1 Center for the Study of Complementary and Alternative Therapies, University of Virginia School of Nursing, Charlottesville, Virginia, USA; 2 University of Virginia School of Nursing, Charlottesville, Virginia, USA; 3 Center for the Study of Complementary and Alternative Therapies, University of Virginia School of Nursing, Charlottesville, Virginia, USA; 4 University of West Georgia School of Nursing, Carrollton, Georgia, USA; 5 Division of Hematology and Oncology, Department of Medicine, University of Virginia Medical Center, Charlottesville, Virginia, USA; 6 Center for the Study of Complementary and Alternative Therapies, University of Virginia School of Nursing, Charlottesville, Virginia, USA

**Keywords:** Acute myelogenous leukemia, Therapeutic massage, Complementary health-enhancing approaches, Complementary and alternative medicine, Integrative medicine

## Abstract

**Objective:**

Cancer treatment is reported to be stressful, and patients diagnosed with hematologic cancers often exhibit higher levels of anxiety and emotional distress than individuals with other malignancies. Management of these symptoms in patients with hematologic cancer presents significant challenges, as many of them are in and out of the hospital while undergoing high dose chemotherapy. Oncology patients use complementary modalities such as therapeutic massage in an attempt to alleviate disease and treatment-related symptoms, including anxiety and emotional distress. In the current study, the feasibility of a novel massage intervention delivered over the continuum of care, as well as assessment of the immediate and cumulative effects of massage, was examined in patients with acute myelogenous leukemia.

**Methods:**

A mixed-methods, unmasked, prospective, randomized study was conducted with two groups: a usual care alone control group and a massage therapy intervention plus usual care group.

**Results:**

Significant improvements in levels of stress and health-related quality of life were observed in the massage therapy group versus the usual care alone group, after adjusting for anxiety level, including both immediate and cumulative effects of massage.

**Conclusions:**

While the findings of the current study regarding acceptability, feasibility, and potential efficacy of therapeutic massage as a complementary health-enhancing intervention in patients diagnosed with acute myelogenous leukemia are very promising, the relatively small size of the study sample limits generalizability.

## Introduction

Cancer treatment is reported to be stressful, resulting in treatment-related symptoms that include depression, anxiety, fatigue, sleep disturbances, and pain. Some patients even describe the aggressive treatment of cancer as more distressing than the cancer itself [1]. Anxiety and depression are prevalent at high levels prior to and following the successful treatment of cancer [2–7]. Patients diagnosed with hematologic cancers such as acute myelogenous leukemia (AML) often exhibit higher levels of anxiety and emotional distress than individuals with other malignancies and struggle to adjust to all that is happening to them in the weeks following diagnosis [4,5,8]. Management of symptoms in patients with hematologic cancer presents significant challenges, as many of these patients are in and out of the hospital while undergoing high dose chemotherapy.

## Background Literature

AML represents approximately one-third of all leukemia diagnoses and can rapidly lead to the loss of normal hematopoietic function [9]. Subsequent bleeding and infectious complications may result in death within weeks to months of the clinical presentation of AML [10]. Patients frequently present with fatigue, hemorrhage, fever, anemia, bruising, lymphadenopathy, and infections [11], which generally lead to immediate and aggressive medical treatment delivered in three phases: induction, consolidation, and maintenance therapies [10]. Induction chemotherapy regimens have changed minimally over the last 20 years [12], although researchers continue to investigate whether or not intensification of induction therapies may actually decrease the chance of relapse [13]. Induction chemotherapy generally involves a week-long hospitalization for treatment, following which patients return home, although some are frequently readmitted with fevers and infections over the next month. Patients return to the hospital for multiple cycles of post-remission chemotherapy of 5 to 6 days duration each month for 3 to 4 months during the consolidation and maintenance stages of treatment. Many patients (50–70%) receive complete remission with current chemotherapeutic protocols [12].

Leukemia symptoms are often difficult to manage by conventional medicine alone [14–19], and deficits in physical and social/occupational health-related quality of life (HQoL) may continue a year or more following treatment [2]. The challenging array of symptoms and physical, emotional, and behavioral stressors impair HQoL both prior to and following treatment [7,20–22]. Stress exacerbates symptoms associated with AML [22], negatively influences treatment outcomes, and has a considerable bearing on patients and their families [14,19,23,24]. In some instances, pharmacological interventions to alleviate symptoms may not be indicated because of the added burden of drug clearance on the liver and kidneys. Patients with cancer may have underlying hepatic and renal impairments that may result in accumulation of the administered chemotherapeutic drugs and psychotropics, and thus elevated plasma drug levels could occur [25,26].

Complementary health-enhancing approaches are popular with the public but less so with some healthcare professionals, in part because of a lack of standardized methodology for assessing outcomes and treatment efficacy [27]. Nonetheless, manual therapies such as therapeutic massage have been shown to be efficacious in reducing perceived psychological stress and can be used when other modalities of stress reduction such as exercise, meditation, cognitive/behavioral psychotherapy, or guided imagery may not be feasible [27].

Massage has been practiced for centuries to promote and restore health [28], and, given the emergence of integrative healthcare, many patients with cancer have reported the use of this complementary modality to reduce disease and treatment-related symptoms that impair functional status and improve HQoL [29–31]. Studies using massage interventions in oncology patients indicate that massage may alleviate a wide range of symptoms, including pain [32–34], nausea [35], anxiety [33,35,36], depression [32,36], anger [36], distress [35], and fatigue [35], with patients experiencing significant increases in short-term HQoL [32] after receiving massage. However, methodological issues concerning the minimization of bias, the blinding of evaluators, the use of suitable control interventions, examination of the immediate and cumulative effects of massage, and the feasibility of the continuity of massage therapy across the continuum of care have not been adequately addressed. Additionally, controlled longitudinal studies documenting the effects of massage among patients with hematologic cancers and the potential benefits of massage during chemotherapy in this population are still lacking.

Thus, the current feasibility study tested the implementation of a novel 7-week complementary health-enhancing program of massage therapy for patients diagnosed with AML to collect preliminary data needed to examine accrual, attrition, patient acceptance, and any potential limitations of the massage therapy intervention and to examine the immediate and cumulative effects of the massage therapy intervention over time on HQoL and treatment-related symptoms (e.g., anxiety, stress, pain, fatigue, and nausea/vomiting).

## Methods

### Study design and subjects

A mixed-methods, unmasked, prospective, randomized study was conducted with two groups: a usual care alone control group (UC) and a massage therapy intervention plus usual care group (MT). Usual care alone consisted of the standard treatment protocol for AML: induction, consolidation, and maintenance therapy. Chemotherapy regimens differed among participants based on cancer cell type. The most commonly used regimen was 7 days of cytosine arabinoside (AraC) plus an anthracycline, most often idarubicin, on the first 3 days.

The study procedures were approved by the University of Virginia Cancer Protocol Review Committee and the Institutional Review Board for Health Sciences Research. Potentially eligible study participants were identified in the oncology-hematology clinic at the University of Virginia Health System as patients who presented for evaluation and diagnosis of AML or were admitted to the hospital for treatment. Before screening for inclusion and exclusion criteria, persons were consented and enrolled in the study. Thus, the study was explained to the potential candidates and those interested in the study were consented and screened on inclusion and exclusion criteria. Inclusion criteria included: (1) a diagnosis of AML; (2) age 18 years or older; (3) able to understand the consent form (written in English at a grade 5–7 level); (4) a platelet count ≥15,000/mm3; (5) able to be contacted by telephone; and (6) willing to abide by the proposed protocol and complete the assessments. Criteria for exclusion were: (1) regular use of massage (≥1 massage/month for 3 months) prior to enrollment in the study; (2) current thrombosis or phlebitis; and (3) persistent anxiety or depression although treated with medication, which could potentially negatively affect study outcome measures. Following completion of the consent form and baseline assessments, participants were stratified by gender because of potential differences between men and women in response to massage [37,38] and randomly assigned with probabilities in a ratio of 1:1 to one of the two treatment groups. Randomization was accomplished using a computer-generated assignment list and was masked (using envelopes). That is, the next available assignment number was not revealed until it was required. Participants in both the UC group and the MT group received a monetary research incentive of $50 for the return of baseline and each weekly assessment, for a total of $400 at study completion.

### Massage procedure

Various types of massage reduce distress, anxiety, pain, fatigue, and nausea in patients undergoing conventional cancer care [39–42]. The current study used gentle Swedish massage, to which previous patients with hematologic cancer had been receptive during hospitalization [35,41–43]. Participants in the MT group received 50 minutes of Swedish massage, which included primarily effleurage (long gliding strokes) and petrissage (gentle kneading-like strokes), three times per week for 7 weeks, for a potential of 21 massages for each participant. The massage protocol was designed to mimic the experience of massage in a non-research setting. Because patients receiving massage report a carryover effect in the day or two following a massage session, an intervention period of three times per week separated by at least one day was chosen to maximize the potential benefits of the intervention. It was assumed that multiple weeks would intensify the positive effects of the massage intervention. Additionally, in this study, the investigators were interested in the immediate as well as the cumulative effects of massage over time in the population of patients with AML; thus, an intervention of three massages per week for 7 weeks provided data that would allow for analyses to address both of these effects.

Although some participants already had a central line in place, the first massage occurred prior to the placement of the participant’s central line whenever possible. The remaining massages were coordinated with participants’ scheduled hospital care and appointments at the oncology-hematology clinic as well as other outpatient appointments, with efforts made not to give massages on consecutive days. During periods when participants were at home between phases of treatment, they received three weekly “in-home” massages to provide continuity of care during the outpatient phases of treatment and to lessen any unnecessary participant burden (e.g., travel to receive the massages). Each participant was assigned the same therapist for all “in-home” massage sessions and a therapist at the medical center for all inpatient massages (all therapists were female). Therapists talked weekly with the study coordinator, and the clinic staff communicated with the study coordinator if a participant had any conditions (e.g., developing phlebitis, shingles) that would preclude massaging affected areas of the body. Massages were deferred if the participant had a platelet count less than 15,000/mm3. Therapists avoided and documented body areas of concern; other areas were massaged and documented on a massage checklist. A standardized, fragrance-free non-allergenic massage cream was used for all massage sessions. Massages were given without any accompanying music to decrease the risk of a confounding variable on stress measures.

For participants randomized to the MT group, a local community massage therapist was identified in the participant’s home area so that staff training, scheduling, and preparations could begin for home massages. All therapists were certified by the National Certification Board for Therapeutic Massage and Bodywork as well as by the Virginia Board of Nursing, the governing body for massage therapy in the Commonwealth of Virginia. All therapists completed the Institutional Review Board online training for protection of human subjects against research risks, reviewed study information, and completed a 3-hour training session specific to the massage protocol, hospital environment, and cancer treatment. The therapists were paid for providing massages.

### Massage protocol

The massage table and any items brought into the participants’ homes or hospital rooms were cleaned and treated with a germicide prior to any contact with participants. The massage therapist followed infection control policies and procedures pertinent to each participant to ensure that participants were not exposed to potential pathogens. If a participant was placed on contact isolation, the massage therapist wore surgical latex gloves, or non-latex if the participant or massage therapist was allergic to latex, to comply with hospital policy. The therapist entered the participant’s room, greeted the participant, and ensured privacy. After obtaining feedback about symptoms being experienced and completing hand washing, the therapist assessed the participant’s skin integrity for skin breaks, lesions, or intravenous sites. Areas of sensitivity and pain were assessed further, and these areas of concern were avoided and documented on the massage checklist. If a participant reported being “too ill” to receive a scheduled massage on a given day, or had developed a complication, the massage coordinator rescheduled the session for as soon as feasible. Participants were assisted in transferring to a massage table when possible. However, those with major impediments to movement or those who preferred not to move onto the massage table received massages in their hospital beds. Therapists avoided initiating any conversation other than to ascertain comfort level and feedback on massage technique, but responded to direct questions asked of them. This conversational behavior is considered standard for professional therapists during a massage. The therapist provided massage to the head, neck, shoulders (avoiding the central line site), back, abdomen (except on the first session because abdominal massage is initially aversive to many persons), feet, legs, hands, and arms as intravenous lines permitted. Participants were asked to identify areas where the massage therapist might spend a greater proportion of time, but an effort was made to provide each participant with a full-body massage during each session. No deep tissue work was included. The duration of massage was standardized to 50 minutes, unless a patient requested stopping the session early.

### Massage checklist

To evaluate the massage protocol, the investigators developed a checklist to be completed by all therapists before and after each massage. The therapists documented the date, time of day, and length of each massage administered. The checklist assessed treatment adherence and integrity (e.g., deviations from the protocol, environmental barriers to massage in the hospital and home) over the course of the intervention. Before conducting the massage, the therapist noted any changes since the previous massage, how the participant was feeling prior to the massage (relaxation and stress), and any areas to avoid during the massage because of discomfort or at the participant’s request. Following the massage, the therapists asked questions regarding levels of relaxation, comfort, and stress and noted any additional comments made by the participant or the therapist. Community-based therapists mailed or faxed their forms on a weekly basis to the study coordinator, who reviewed the measures and clarified any points in a scheduled weekly telephone call with each therapist.

### Outcome measures

Self-report and objective measures of HQoL were collected from participants in the MT and UC groups weekly throughout the 7-week study. Participants completed and returned demographic data and medical history forms prior to group assignment. All medical information provided by each participant was cross-validated against the participant’s medical record or with the hospital staff. On the days of the first and last massages, the MT group participants responded to several open-ended questions to document the participants’ previous experiences with massage therapy and to evaluate the participants’ perceptions of massage provided during the study. The following questionnaires were completed by all participants at baseline: European Organization for Research and Treatment of Cancer core questionnaire (EORTC-QLQ-C30), the state scale of the State-Trait Anxiety Inventory-Form Y (STAI-Form Y), and the short-form McGill Pain Questionnaire (SF-MPQ) to evaluate the constructs of HQoL, anxiety, and pain, respectively. Participants in the UC group completed the same weekly questionnaires and rating scales as those in the MT group.

### Health-related quality of life (HQoL)

The EORTC-QLQ-C30 reflects the multidimensionality of the HQoL construct for patients with cancer and is one of the most widely used and validated measures of HQoL for these patients [44]. The EORTC-QLQ-C30 includes 30 items comprising five functional scales (physical, role, cognitive, emotional, and social), two symptom scales (fatigue and nausea/vomiting), and the global QoL scale (two items), all of which were used in this study. Higher mean scores on the global scale and the functional subscales represent better function and higher HQoL. No time frame is referenced for these functional questions, although for the remaining questions the participant responds within the context of the last week. Higher means on the symptom subscales represent more symptomatology and lower HQoL. The global health status/QoL is an 8-point scale (1–7), ranging from “Very poor (1)” to “Excellent (7),” and is a primary measure of QoL.

### State anxiety

The STAI [45,46] is the standard instrument to assess anxiety [47]. This instrument measures feelings of apprehension, tension, nervousness, and worry. The state scale consists of 20 items, rated on a 4-point Likert scale, ranging from “Not at all (1)” to “Very much so (4),” representing low to high anxiety for how a person “feels right at this moment” or emotionally responds to a stressful situation [48]. The score ranges from 20 to 80, with 20 to 39 representing low anxiety, 40 to 59 moderate anxiety, and 60 to 80 high anxiety, with higher scores reported under stressful conditions [48].

### Perceived stress, numeric rating scales (NRS)

Stress was measured using an 11-point NRS. Participants rated their stress, feeling of relaxation, and comfort on a 0 to 10 scale “at this moment” immediately before and after each massage session. For example, the NRS for stress ranged from 0 (no stress) to 10 (extremely stressed). The NRS is a simple, yet sensitive measure of subjective phenomena and has been used successfully in a previous study of the effects of massage [49]. Validity and reliability of the NRS as a measure of symptoms has been demonstrated [50,51].

### Pain

The SF-MPQ [52] measures sensory and affective aspects of pain and contains sensory descriptors (throbbing, shooting, stabbing, sharp, cramping, gnawing, hot-burning, aching, heavy, tender, and splitting) and affective descriptors (tiring-exhausting, sickening, fearful, and punishing-cruel). The descriptors are ranked from 0 to 3 (none, mild, moderate, and severe) [52].

### Data analysis

Quantitative data were analyzed using SPSS v.19. Descriptive statistics (means and standard deviations for continuous variables, and frequencies and percentages for categorical variables) were calculated on demographic and baseline outcome variables. Differences in demographic and baseline outcome measures between groups (MT vs. UC) were tested using independent t-tests for continuous variables and chi-squares for categorical variables.

Separate multilevel models were used to estimate differences in the slopes between the groups for each of the outcome variables. Model parameters were estimated by restricted maximum likelihood, and the choice for model fit between the unconditioned models (no covariates) and the conditioned models (with covariates) was determined by Akaike’s information criterion (AIC). Random intercepts and slopes were estimated from the data. The models estimated change for participants as well as the differences in average slopes for each group (MT vs. UC). The conceptual model used in this study indicated that stress and state anxiety should be included as covariates (the only exception was when stress was the dependent variable, in which case state anxiety was the only covariate). Because stress and state anxiety were expected to vary over the study given the AML treatment regimen, these were used as time-varying covariates in the conditioned models.

## Results

The sample consisted primarily of White participants, almost equally split by gender, and the majority were married and on average had a high school education or slightly above. At baseline, depression and anxiety averages were moderate to high, while sensory pain and nausea/vomiting levels were fairly low. Differences in demographic and baseline study variables between the UC and MT groups were tested (see Table 1).

The only group difference was in baseline fatigue, with the MT group having higher levels of fatigue at baseline than the UC group (p = 0.04).

### Accrual, retention, and attrition

Of the 43 individuals with AML who were identified in the oncology-hematology clinic when they presented for evaluation and diagnosis or upon admission into the hospital, 20 (46.5%) were enrolled. Of the remaining 23 individuals, 13 did not meet the inclusion criteria and 10 declined to participate. Reasons for declining to be in the study included a preference not to be touched, reluctance to commit to filling out the study questionnaires, and a fear that massage might interfere with their hospital schedules and treatment.

**Figure 1:**
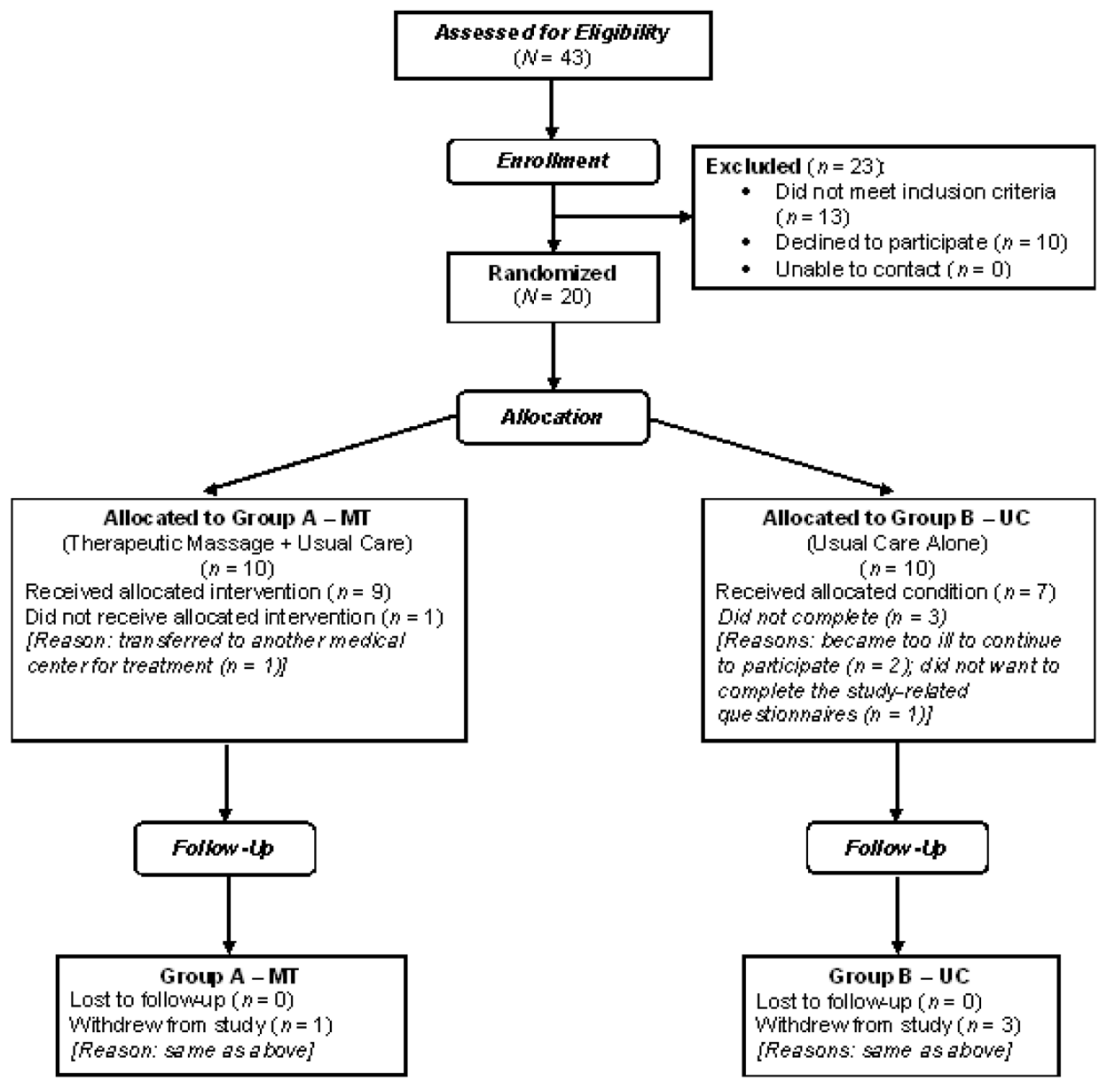
CONSORT flow diagram.

Attrition is shown in Figure 1, which reveals that four individuals did not complete the study (three in the UC group and one in the MT group). The reasons given for not completing the study included becoming too ill to continue (2 in UC), transfer to another medical center (1 in MT), or not wanting to complete the study-related questionnaires (1 in UC). Study completers (16) and non-completers (4) did not differ on demographic or baseline study variables except age; non-completers were older (63.8 years ± 5.1) than completers (49.5 ± 15.9). The only non-completer in the MT group withdrew after baseline assessments. Of the 3 non-completers in UC, one withdrew after week 2, one after week 3, and one after week 5 (none of the 3 had missing weekly data up until the time of withdrawal).

When considering the 16 participants who completed the study, weekly data were rarely missing for the 8 weeks (baseline and 7 weeks of the intervention period). Of the completers in the UC group, only one participant had missing weekly data and only at one time point. Of the completers in the MT group, 3 had missing weekly data (2 missed only 1 week and 1 missed 3 weeks). Thus, it seems that collecting the weekly data was feasible in this sample.

In the MT group, the average number of massages for all participants was 15.8 (SD = 5.0), with a median of 17. Six participants in the MT group missed at least one massage. The major reason for missing a massage was because of low platelet counts (stipulated as <15,000/mm3 in the study protocol and representing 62% of the missed massages), while the other reason was not wanting to be touched or not feeling well enough for a massage on a particular day (38% of the missed massages). Thus, it seems feasible to perform most massages in participants with AML, knowing that some will be missed because of the effects of the illness and/or AML treatment regimens rather than the massage protocol/intervention itself.

Because the goal of the study was to test the feasibility of massage as an intervention in persons undergoing treatment for AML, testing for statistical significance was examined but was not the primary goal. Additionally, given that the investigators were interested in both the cumulative and immediate effects of massage, only the completers were included in the remainder of the statistical analyses.

### Perceived benefits of massage

Following completion of the final massage of the study, participants in the MT group were asked about their perceptions of the benefits of massage over the course of the study. All participants in the MT group indicated the benefits of massage included stress reduction, increased comfort, and increased relaxation. One participant described the massages as “always very warm and comforting,” remarking that “any human touch is good” and that “anyone could benefit.” Another participant indicated that he planned to use the money he received for completing the study to continue receiving massages. Participants described receiving massage in the outpatient setting as “better than in [the] hospital,” because they felt “more relaxed,” thus preferring massages “in home” over those in the inpatient setting.

### Perceived barriers to massage

Participants in the MT group also were queried about perceived barriers to massage over the course of the study. In cases when gloves were used during the massage for infection control purposes, participants remarked that the “hands felt rougher” and “not as gentle” but did not indicate that the use of gloves hindered the beneficial effects of massage overall. Several participants were fearful of or unable to lie in a prone position on the massage table. The clinical environment of the inpatient setting was not conducive to relaxation in some instances, and one participant suggested the addition of music during the massage to further promote relaxation, though this participant understood this would be a confounding factor in the study design.

### Accommodations to the massage protocol

Accommodations and modifications to the massage protocol were noted on the massage checklist. Areas of the body that participants most frequently requested not to be massaged were the abdomen and the scalp, which were described by a few participants as “too sensitive,” particularly the head given hair loss related to chemotherapy. In several instances, massage time was decreased in the inpatient setting because of interruptions such as telephone calls, ultrasounds or other procedures, and meals, or because participants were not feeling well. During these instances, massage time most often was decreased by about 5 minutes. In the home settings, no deviations in the standardized time of the massage protocol were noted. Accommodations were made during the massage to avoid the central line, and gloves were used in cases of participants being in contact isolation because of VRE, MRSA, or C. difficile infection. On several occasions, massages were rescheduled because of decreased platelet counts (<15,000/mm3 ), while some massages were missed because of repeatedly low platelet counts. Massages also were rescheduled when participants did not feel well enough for massage at the moment or when, as in three instances, participants stated that they “[did] not want to be touched” that day. One massage in the inpatient setting was rescheduled because the participant had a high fever. Several massages were conducted with participants lying on their side because of discomfort while lying supine. Two massages were conducted with masks worn because of airborne precautions. In one instance of a participant’s remote rural home setting, no local massage therapist was located and massages were conducted in the home setting by the study coordinator, who is a certified massage therapist.

Conducting the massage intervention in the home setting proved feasible and effective given that the massage therapists did not report any difficulties in providing massage in the home environment overall and the participants noted that they could feel more relaxed in the home setting. Scheduling of in-home massages did pose some challenges given the difficulty in finding a community-based therapist in several of the remote rural areas as well as coordinating the in-home massage with a time that was best for the participant.

### Symptom outcomes

**Figure 2:**
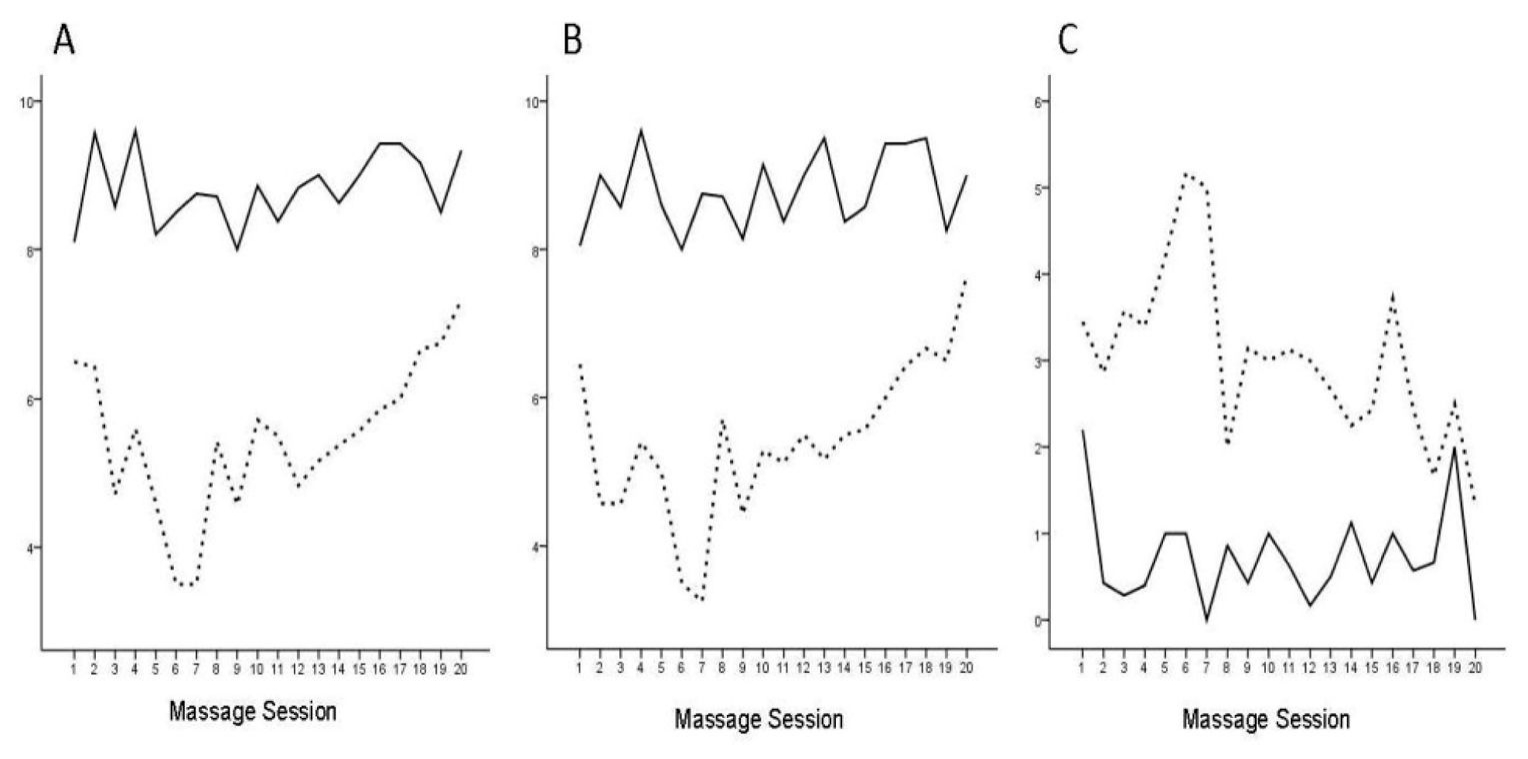
Pre- and post-treatment effects of massage. Immediate and cumulative effects of massage therapy sessions are presented, with mean scores for (A) comfort, (B) relaxation, and (C) stress. Pretreatment mean scores are represented by dotted lines; post-treatment mean scores are represented by solid lines.

For those in the MT group, differences in comfort, relaxation, and stress occurred after massage. The immediate effects of massage are shown in Figure 2, displaying the increases in comfort and relaxation, and the decreases in stress from pre- to post-massage. This figure also displays the cumulative effects of massage, as can be seen by the increasing values for pre-comfort and pre-relaxation, as well as the decreasing values of stress over time, both before and after the massage sessions.

Testing statistical significance was not the primary aim of this feasibility study; however, results were examined for significance and trends. When treated as a dependent variable, state anxiety did not change significantly over time, nor did the groups differ over time. In the unconditioned model of stress (Table 2), there was not a significant difference between the groups over time (p = 0.135). However, after controlling for state anxiety over time, a significant difference between the groups (p = 0.041) was found: the MT group had a statistically significant decrease in stress over time compared to the UC group, whose slope did not change.

The AIC was significantly reduced in the conditioned model for stress compared to the unconditioned model (Table 2), indicating that the conditioned model was a better fit. When considering the unconditioned model of HQoL, there was not a significant difference between the groups over time (p = 0.125). However, after controlling for stress and state anxiety over time, a trend toward a difference between the groups (p = 0.069) was found: the MT group had an increase in HQoL over time compared to the UC group. Given that no differences were found over time or between the groups in the symptoms of sensory or affective pain, fatigue, or nausea/vomiting, these data are not shown.

## Discussion

The study results reveal that it is feasible to provide massage across the continuum of outpatient, inpatient, and in-home settings to patients undergoing treatment for AML while making accommodations for individual patient needs. A novel aspect of the current study was the delivery of massage therapy in both the acute care and home settings, lending continuity to the intervention over the treatment period. To our knowledge, this is the first published study of therapeutic massage or any other complementary health-enhancing manual therapy to use this approach in any patient population. No participants in the MT group expressed any significant problems receiving massage in-hospital or in their home environments.

Although only 20 of the 43 eligible patients were recruited for this study, previous studies have reported that patients undergoing chemotherapy for cancer are very receptive to the use of massage therapy during both hospitalization and after discharge [35,41,43,53], which is evident in the qualitative responses from participants in the current study. Overall, the participants indicated that the massages “increased comfort,” “reduced stress,” and “increased relaxation.” Several participants noted that the massages had made the hospital stay more tolerable, and one participant planned to use the monetary research payment provided to get additional massages once the study had concluded. Three of the four participants who withdrew from the UC group did so because of illness or transfer to another medical center, while only one dropped out because of study-related activities (i.e., questionnaire completion). This indicates the feasibility of the intervention given that the majority of the attrition in the UC group was not study-related and the remaining participants did not find the number of questionnaires excessive. While glove use was required for some massages, overall use of gloves did not interfere with the massage therapists providing comforting massages; however, several participants who received massages from a therapist wearing gloves commented that the therapists’ hands felt a bit rough and that those massages were not quite as comfortable as when gloves were not needed.

Anxiety is a common symptom among patients with cancer in general [5]. Patients with AML undergoing treatment experience high levels of anxiety and distress, with an estimated 20% to 35% reporting increased anxiety during the induction and consolidation phases of chemotherapy [4,5]. These levels of anxiety and distress have been shown to decrease over the course of treatment, with higher levels seen while patients with AML are receiving intravenous chemotherapy [5]. In the current study, stress significantly decreased over time in the MT group versus the UC group after controlling for state anxiety (Table 2). Additionally, a trend toward a statistical improvement in HQoL was found in the MT group compared to the UC group when controlling for both stress and anxiety. No statistically significant change in sensory or affective pain, fatigue, or nausea/vomiting was observed following the massage intervention. Additionally, the instruments used to collect these outcomes may not have been sensitive enough to detect small changes in pain, fatigue, or nausea/vomiting levels over time.

Though the primary aim of the current study was to assess the feasibility of the novel intervention protocol, the small sample size limited the statistical power in analyzing the data for significant effects on disease and treatment-related symptoms and HQoL. The variability in the number of massages received by the participants in the study also is a limitation when looking at the effects of massage over time. There was no follow-up period to examine the level of symptoms following the intervention and treatment period. Long-term survivors of leukemia have shown persistently high levels of anxiety and distress following chemotherapy [4]. Despite these limitations, statistical differences and trends were observed between the MT and UC groups.

The current findings provide longitudinal data demonstrating the beneficial effects of massage in patients with AML. Given the small sample size and the limited generalizability of the findings of the current study, larger trials with adequate statistical power should be conducted to examine the efficacy of therapeutic massage in this patient population. Future trials examining the potential mechanisms of action of massage therapy as well as other manual therapies are likely to advance the science in this field and enhance the value of massage in the quality of care provided to patients. The words of one participant, who noted, “I believe the whole massage process promotes healing--physical, mental, and....,” perhaps provide the best summarizing statement for this study.

## Figures and Tables

**Table 1:** Group differences on demographics and baseline study variables

	UC		Massage		p-value
	n	%	n	%	χ^2^
Gender					0.95
Male	4	57.1	5	55.6	
Female	3	42.9	4	44.4	
Marital status					0.38
Married	6	85.7	6	66.7	
Other	1	14.3	3	33.3	
Race					0.85
Nonminority	6	85.7	8	88.9	
Minority	1	14.3	1	11.1	

	Mean	SD	Mean	SD	t-test
Age	46.3	14.3	52	17.5	0.5
Education	13.3	2.2	12.1	5	0.58
State anxiety	40.6	13.4	35.6	9.3	0.39
Stress	1.4	2.2	4.7	3.6	0.06
Sensory pain	7.3	5.3	4.6	4.4	0.28
Affective pain	2.3	2.6	1.4	2	0.48
Fatigue	42.6	26.7	72.8	24.3	0.04
Nausea/vomiting	22.2	25.1	18.5	25.6	0.79
HQoL	52.8	22.8	39.8	28.8	0.37

**Table 2:** Analysis of changes in stress and HQoL

	Unconditioned model			Conditioned model		
	Estimate	SE	p-value	Estimate	SE	p-value
Stress						
Intercept	2.98	0.66	<0.001	−2.29	1.13	0.063
Time	0.09	0.16	0.584	0.2	0.13	0.171
Group	1.98	0.89	0.028	2.41	0.93	0.011
Time by group	−0.33	0.22	0.135	−0.38	0.18	0.041
State anxiety				0.12	0.02	<0.001
AIC	574.1			553.5*		
HQoL						
Intercept	71.86	8.85	<0.001	114.95	18.5	<0.001
Time	5.62	2.95	0.077	4.56	2.72	0.116
Group	−12.12	11.78	0.307	−16.24	12.38	0.193
Time by group	6.09	3.93	0.125	6.67	3.62	0.069
Stress				−0.34	1.52	0.825
State anxiety				−0.96	0.4	0.019
AIC	1197.9			1188.60*		

HQoL = Health-related quality of life; AIC = Akaike information criterion

* Significant decrease in AIC in conditioned model compared to the unconditioned model
